# Supplementary material for: Evaluation of the Elements of Short Hairpin RNAs in Developing shRNA-Containing CAR T Cells
Source: Cancers (Basel). 2023 May 20;15(10):2848. doi: 10.3390/cancers15102848 (PMC10216594; doi:10.3390/cancers15102848)
Supplement: Supplementary file 1 [file cancers-15-02848-s001.zip › cancers-2392736-supplementary.pdf]

Supplemental Figures

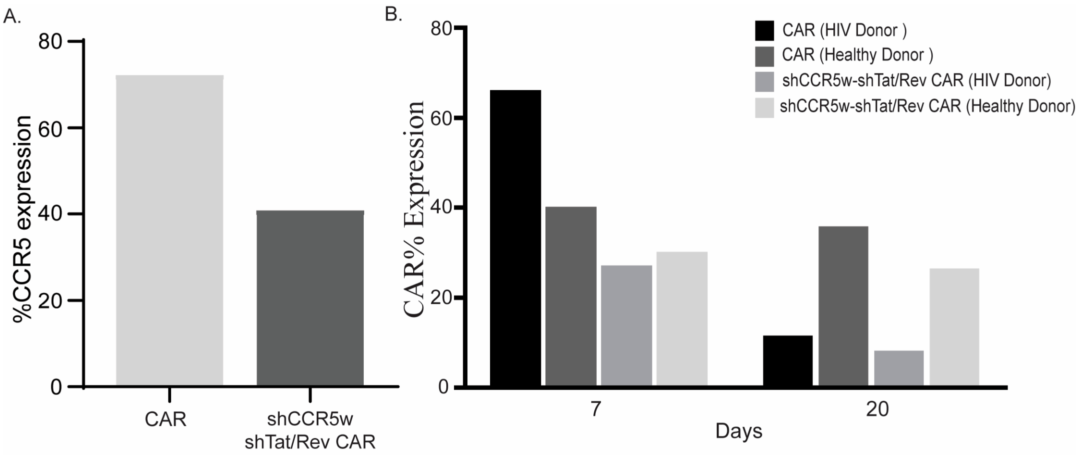

Figure S1. Evaluating shCCR5 function and CAR expression. (A) qPCR analysis of CCR5 in CCR5-expressing MAGI cells when comparing shCCR5w-shTat/Rev CAR with conventional CAR (n = 1). (B) Comparison of CAR expression of healthy donor to PLWH T cells for conventional and shRNA-containing CAR at day 7 and 20 (n = 1).

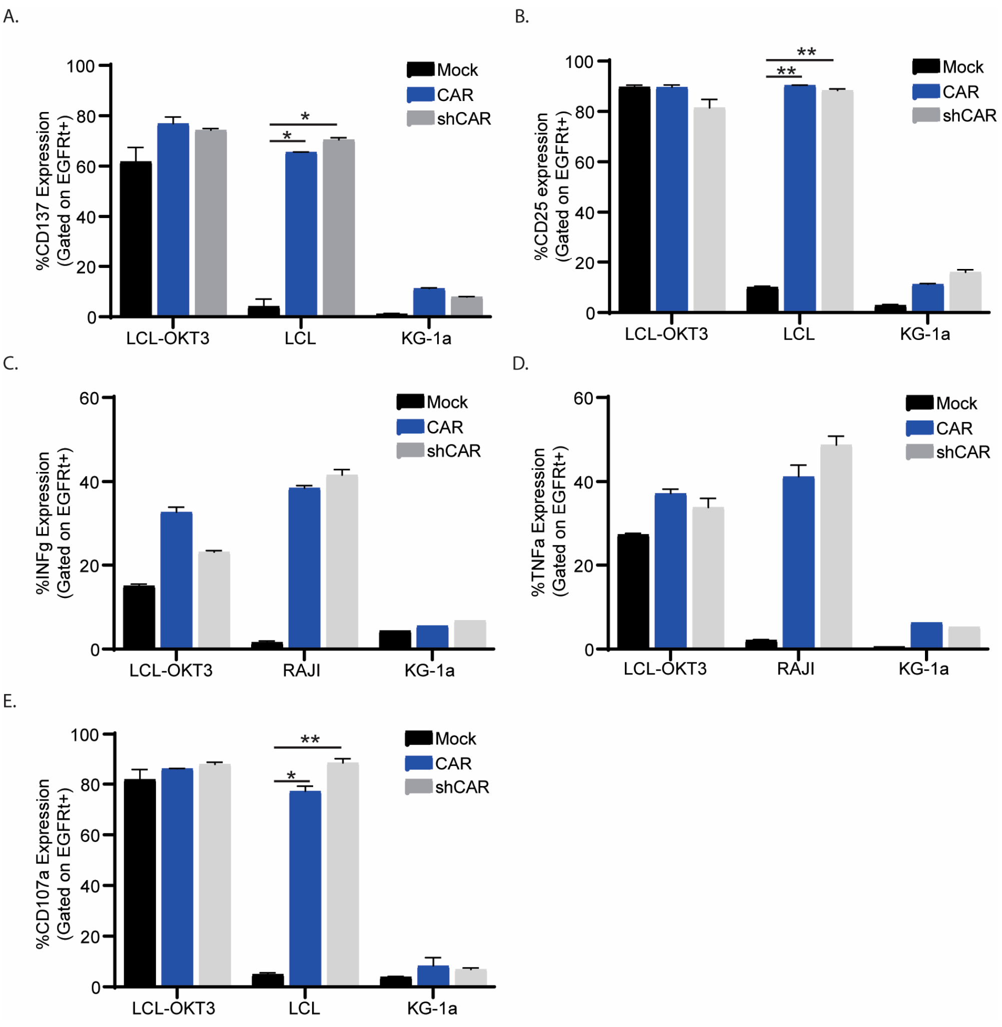

Figure S2. Functional analysis of shHPRT CAR compared to conventional CAR. shHPRT CAR has similar activation, CD137 (n = 2) (A) and CD25 (n = 2) (B), cytokine production, interferon-gamma (n = 2) (C) and TNF alpha (n = 2) (D), and degranulation production (n = 2) (E) as conventional CAR T cells. LCL-OKT3 is the positive control and KG-1a is the negative control. To ensure accuracy and reliability, each experiment had technical replicates (n). This figure represents 1

healthy donor of the 3 healthy donors that were interrogated. The symbols ** and * represent a p value 0.001 and 0.005, respectively.

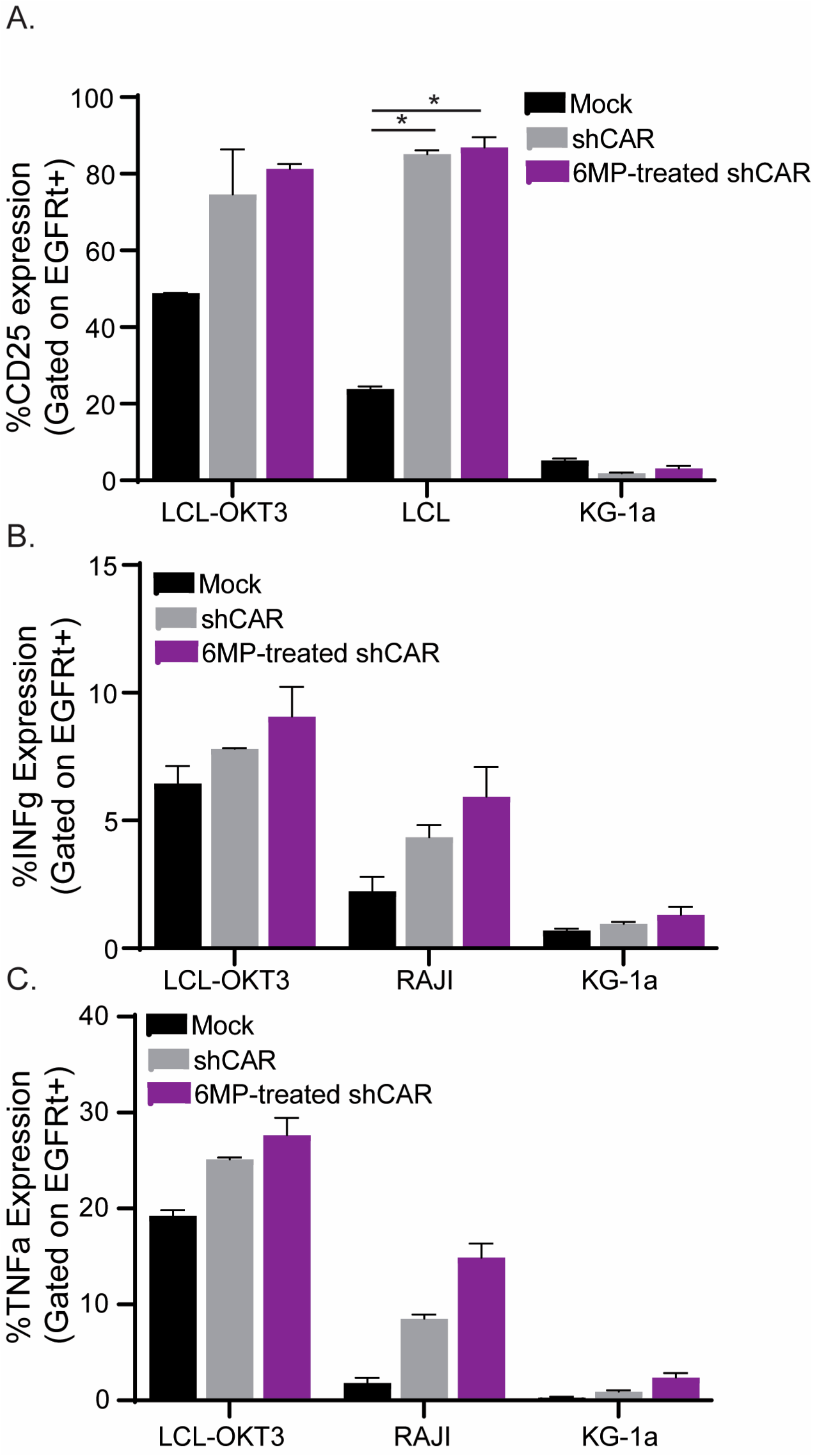

Figure S3. Functional analysis of post-enrichment shHPRT CAR compared to untreated shHPRT CAR. shHPRT CAR with or without 6MP treatment had similar CD25 (n = 2) (A), interferon-gamma (n = 2) (B) and TNF alpha (n = 2) (C). LCL-OKT3 is the positive control and KG-1a is the negative control. To ensure accuracy and reliability, each experiment had technical replicates (n). This figure represents 1 healthy donor of the 3 healthy donors that were interrogated. The symbols * represent a p value 0.005, respectively.

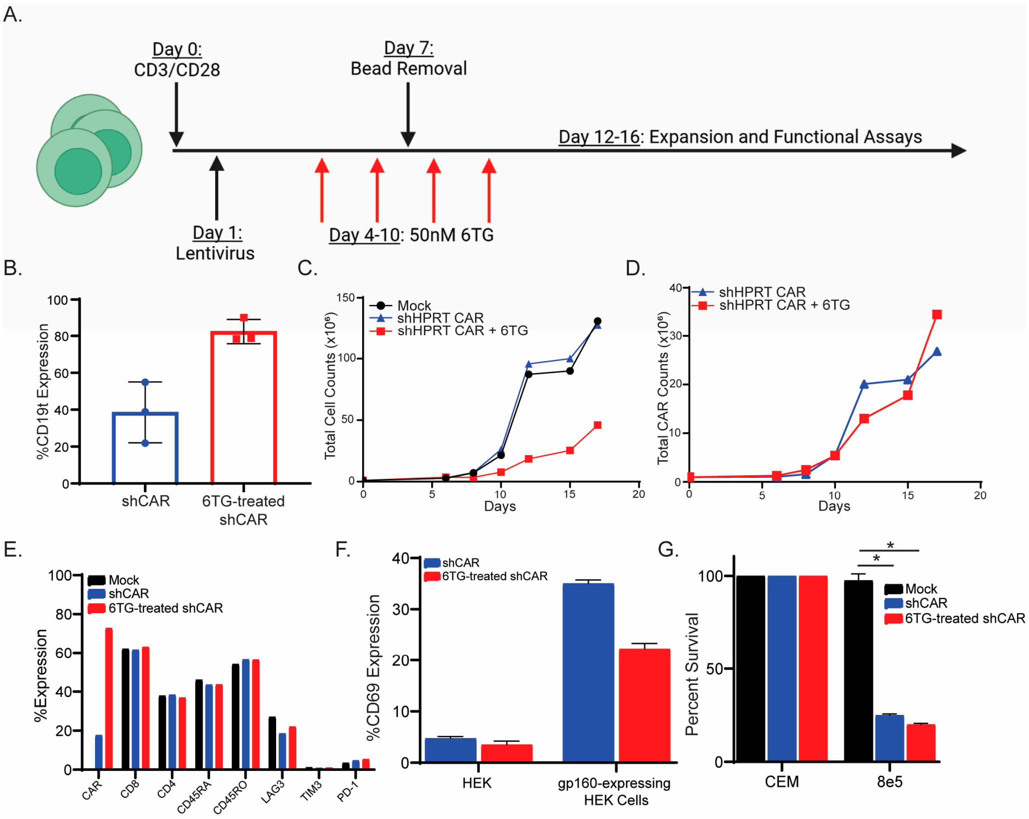

Figure S4. 6thioguanine enrichment of shHPRT CAR did not impact growth or function. (A) Schema of 6thioguanine enrichment of shHPRT CAR T cells. (B) 6thioguanine enrichment of shHPRT CAR (*n* = 3 healthy donor). Growth curve of 6thioguanine treated shHPRT CAR T cells as compared to mock and untreated shHPRT CAR for total T cells (C) and total CAR (D). Phenotype analysis of 6thioguanine treated shHPRT CAR T cells when compared to untreated. Functionality analysis, CD69 activation (*n* = 2) (F) and killing (*n* = 2) (G), of 6thioguanine, treated and untreated shHPRT CAR T cells. To ensure accuracy and reliability, each experiment had technical replicates (*n*). This figure represents 1 healthy donor of the 3 healthy donors that were interrogated. The symbols * represent a *p* value 0.005.
